# Interleukin-9 Promotes Pancreatic Cancer Cells Proliferation and Migration via the miR-200a/Beta-Catenin Axis

**DOI:** 10.1155/2017/2831056

**Published:** 2017-03-02

**Authors:** Bangli Hu, Huang Qiu-lan, Rong-e Lei, Cheng Shi, Hai-xing Jiang, Shan-yu Qin

**Affiliations:** Department of Gastroenterology, The First Affiliated Hospital of Guangxi Medical University, Nanning 530021, China

## Abstract

*Background*. Both IL-9 and miR-200a are involved in the pathogenesis of cancers; however, the role of IL-9 in pancreatic cancer and the possible underlying mechanisms remain unknown. The aim of this study was to investigate the effect of IL-9 on pancreatic cancer cells and its interaction with miR-200a.* Methods*. Pancreatic cancer cells (PANC-1 and AsPC-1) were treated with IL-9 and the expression of miR-200a and *β*-catenin in pancreatic cancer cells was measured. *β*-Catenin was examined as a target gene of miR-200a in pancreatic cancer cells. The interaction between IL-9 and miR-200a in pancreatic cancer cells was determined by infecting miR-200a mimics prior to IL-9 treatment and then measuring miR-200a and *β*-catenin expression.* Results*. IL-9 significantly promoted the proliferation, invasion, and migration of pancreatic cancer cells; however, the effect on pancreatic cancer cell apoptosis was insignificant. *β*-Catenin was verified as a target gene of miR-200a in pancreatic cancer cells. Overexpression of miR-200a in pancreatic cancer cells significantly attenuated proliferation and metastasis and reduced *β*-catenin expression. IL-9 treatment of pancreatic cancer cells decreased miR-200a expression and increased *β*-catenin expression. The effect of miR-200a on pancreatic cancer cells decreased following IL-9 treatment.* Conclusions*. IL-9 promotes proliferation and metastasis in pancreatic cancer cells; this effect may partly involve regulation of the miR-200a/*β*-catenin axis.

## 1. Introduction

Pancreatic cancer is a highly lethal malignancy and the fourth most common cause of cancer deaths worldwide. The prognosis of pancreatic cancer remains quite poor, with overall 5-year survival rates of <5%, mostly due to local recurrence and metastasis [1]. Though great progress has been made over the past few decades, the pathogenesis of pancreatic cancers remains largely unknown, especially the mechanism underlying cancer metastasis. miR-200a expression has been reported to be downregulated during the progression of many cancers. miR-200a is able to suppress the epithelial-mesenchymal transition (EMT) process [2–4], which is a critical step in cancer metastasis initiation. In addition, miR-200a has also been shown to suppress cell proliferation and metastasis in several cancers such as pancreatic cancer [5] and hepatocellular carcinoma [6]. More recently, Wu et al. reported that miR-200a suppresses pancreatic cancer cell metastasis via the downregulation of DEK, suggesting that miR-200a may constitute a novel target for pancreatic cancer treatment [7].

IL-9 is a multifunctional cytokine secreted by activated Th2 cells, Th9 cells, and regulatory T-cells [8]. The targets of IL-9 include mast cells, T-cell clones, and B-lymphocytes [8], through which IL-9 plays a crucial role in immune reactions against parasites [9], asthma [10], and lymphoma [11] development. In addition to its role in immune and inflammatory diseases, emerging evidence indicates that IL-9 participates in the pathogenesis of cancers, acting mostly as a cancer development promoting factor, especially in nonsolid tumors [12–14]. The interaction between cytokines and miRNAs during tumorigenesis is receiving increasing attention; examining this interaction is critical to uncovering the mechanisms underlying tumorigenesis. Therefore, this study aimed to investigate the role of the interaction between IL-9 and miR-200a in the modulation of pancreatic cancer progression in order to elucidate the underlying molecular mechanisms.

## 2. Materials and Methods

### 2.1. Cell Culturing

Two pancreatic cancer cell lines (PANC-1 and AsPC-1 cells) and one normal pancreatic cell line (HPDE6-C7) were purchased from the ATCC and cultured in Dulbecco's modified Eagle's medium (DMEM; Gibco BRL Co. Ltd., USA) supplemented with 10% fetal bovine serum (FBS; Gibco), 100 U/ml penicillin, and 100 mg/ml streptomycin. Culture medium was replaced every 24 h unless otherwise stated and cells were incubated at 37°C with 5% CO_2_.

### 2.2. RNA Extraction and Quantitative Real-Time PCR (qRT-PCR)

Total RNA plus miRNAs were isolated from cultured cells or tissue using the mirVana miRNA isolation kit (TaKaRa, Dalian, China), reverse transcribed, and then subjected to SYBR Green-based RT-PCR analysis (TaKaRa). qRT-PCR assays for miR-200a and *β*-catenin were performed using the PrimeScript RT Reagent Kit (TaKaRa), SYBR Green miRcute miRNA Real-Time PCR Kit (QIAGEN, Hilden, Germany), SYBR Green Real-Time PCR Master Mix, and Premix Ex Taq (TaKaRa) according to the manufacturers' protocols.

The assays were performed with the following primers: miR-200a 5′-CGT AAC ACT GTC TGG TAA CGA TGT-3′; U6 5′-CGC AAG GAT GAC ACG CAA ATT CGT-3′; *β*-catenin forward 5′-GAA ACG GCT TTC AGT TGA GC-3′ and reverse 5′-CTG GCC ATA TCC ACC AGA GT-3′; and GAPDH forward 5′-CGG ATT TGG TCG TAT TG-3′ and reverse 5′-GAA GAT GGT GAT GGG ATT-3. These primers were purchased from Sangon Biotech (Shanghai, China). miR-200a level was normalized to U6, and *β*-catenin level was normalized to GAPDH. Gene expression was analyzed using the 2^−ΔΔCT^ method. At least three independent experiments were conducted for each experimental condition.

### 2.3. Western-Blot Analysis

Protein was collected from 2 × 10^6^ cells treated with IL-9 for 48 h using a lysis buffer containing protease and phosphatase inhibitors (Sigma-Aldrich, St. Louis, MO, USA). Cell lysates were centrifuged at 14,000 rpm at 4°C for 15 min. Protein samples were mixed with 5x sample buffer (4 : 1 ratio) and heated at 95°C for 10 min and then separated on 10% sodium dodecyl sulfate-polyacrylamide gels. Following electrophoresis for 90 min, the proteins were transferred onto a polyvinylidene fluoride membrane (Merck Millipore, Billerica, MA, USA). The membranes were blocked for 1 h at room temperature with 5% nonfat milk in 0.1% TBS-Tween 20 and then incubated with anti-*β*-catenin (1 : 2000 dilution; Abcam, Cambridge, MA, USA) at 4°C overnight, followed by incubation with LI-COR IRDye 680-labeled secondary antibodies (Rockland Immunochemicals, Gilbertsville, PA, USA) for 1 h at room temperature. Signals were detected with an Odyssey Infrared Imaging System (LI-COR Biosciences, Lincoln, NE, USA) and quantitated using the FluorChem 8900 system (Alpha Innotech, San Leandro, CA, USA).

### 2.4. Cell Proliferation Assay

The PANC-1 and AsPC-1 cells were treated with different concentrations of IL-9 for 48 h. After that, cells were seeded at a density of 10^4^ per well in 96-well plates and the media were changed to without IL-9. No vehicle control was used. Then, the Cell Counting Kit-8 (CCK-8) assay was used to detect cell proliferation according to the manufacturer's protocol; 1 × 10^4^ cells were plated in 96-well microplates, and then 10 *μ*l of CCK-8 (Dojindo, Beijing, China) solution was added to each well and the samples were incubated for 1 h prior to measuring absorbance at 450 nm. Experiments were performed in triplicate.

No vehicle control was used.

### 2.5. Cell Apoptosis Analysis

The preparation of cell apoptosis was similar to the cell proliferation assay. Quantification of apoptotic cells was performed using the FITC Annexin V Apoptosis Detection Kit I (BD Biosciences, Vienna, Austria) according to the manufacturer's instructions. Analyses were conducted with a BD FACSCalibur flow cytometer (BD Biosciences); apoptotic cells were defined as Annexin V-positive cells. Three independent experiments were performed.

### 2.6. In Vitro Migration and Invasion Assays

Cell migration and invasion abilities were tested using the Transwell assay. For the migration assay, PANC-1 cells were placed in the upper chamber of each insert (Corning Inc., Corning, NY, USA). For the invasion assay, the cells were placed in the upper chamber of inserts coated with 45 *μ*g Matrigel (BD Biosciences) diluted to 2 *μ*g/*μ*l in DMEM. Medium supplemented with 10% FBS (600 *μ*l) was added to the lower chambers. Following several hours of incubation, the upper surface of the membrane was wiped with a cotton tip and cells attached to the lower surface were stained for 20 min with crystal violet and then rinsed in PBS and subjected to microscopic inspection. Invasion values, obtained by counting eight fields per membrane, represent the average of three independent experiments.

### 2.7. Lentiviral Vector Infection of PANC-1 Cells

In order to upregulate hsa-miR-200a expression, hsa-miR-200a mimic lentivirus and a nonspecific control lentivirus were purchased from the Biological Technology Company (GenePharma, Shanghai, China). The sequences were as follows: miR-200a mimic sense 5′-UAA CAC UGU CUG GUA ACG AUG U-3′ and antisense 5′-AUC GUU ACC AGA CAG UGU UAU U-3′. PANC-1 cells plated in a 6-well plate were infected with the hsa-miR-200a-expressing lentivirus vector. Following 72 h of infection, the cells were observed by immunofluorescence microscopy; >80% of the cells expressed green fluorescence protein (GFP), which confirmed the high infection efficiency of the miR-200a vector in PANC-1 cells.

### 2.8. miR-200a Target Luciferase Reporter Assay

The *β*-catenin 3′-UTR sequences containing the predicted miR-200a binding sites were cloned into psiCHECK2 control vector (C8021, Promega, USA) to generate the plasmid psiCHECK2-WT-*β*-catenin 3′-UTR. The psiCHECK2-MUT-*β*-catenin 3′-UTR was generated from psiCHECK2-WT-*β*-catenin 3′-UTR by deleting the “ACTGTAC” binding site for miR-200a. For the luciferase reporter assay, HEK293 cells were coinfected with the luciferase reporter vectors or miR-200a mimics using Lipofectamine 2000 (Invitrogen, Carlsbad, CA, USA). Luciferase activity was measured following infection for 48 h using the Dual-Luciferase Reporter Assay (Promega, Madison, WI, USA) with a luminometer (Synergy 4 Hybrid Microplate Reader, BioTek, Winooski, VT, USA). Luciferase activity values were normalized to* Renilla* values.

### 2.9. Statistical Analysis

All data were analyzed with SPSS 16.0 software (SPSS Inc., Chicago, IL, USA). Data were expressed as mean values ± standard deviation (SD). Comparisons between two groups were carried out using Student's* t*-test. Comparisons of multiple groups were analyzed using one-way analysis of variance (ANOVA) followed by the LSD post hoc test. Differences were considered to be statistically significant at *p* < 0.05.

## 3. Results

### 3.1. IL-9 Promotes Pancreatic Cancer Cell Proliferation and Metastasis

**Figure 1:**
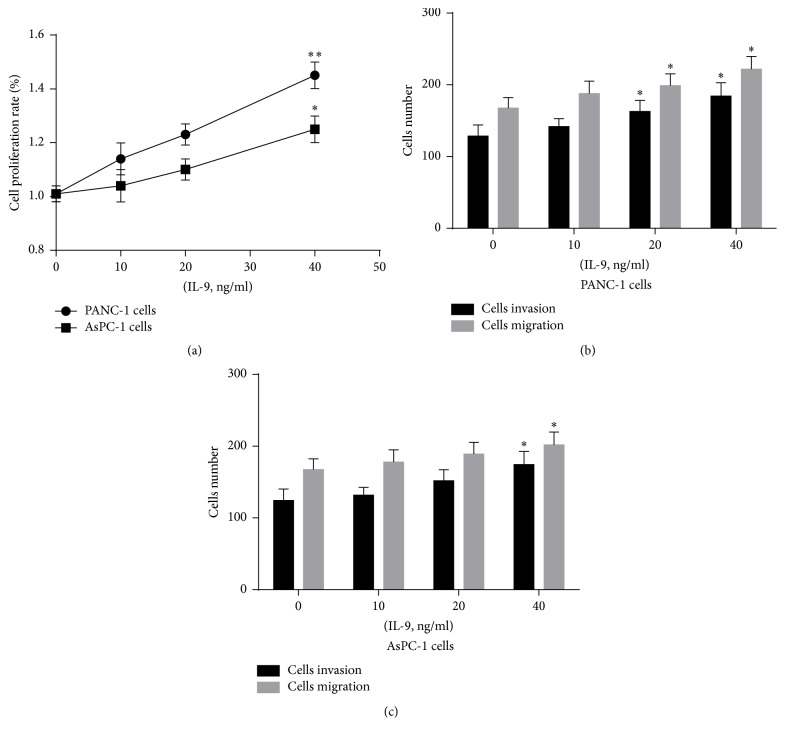
IL-9 promoted pancreatic cancer cells proliferation and metastasis. (a) Proliferation of PANC-1 and AsPC-1 cells was increased with the elevation of IL-9 concentration tested by CCK8 methods. (b)-(c) The invasion and migration rate of PANC-1 and AsPC-1 cells was increased after IL-9 treatment using Transwell assay. Data was expressed as mean ± SD. ^*∗*^*p* < 0.05. ^*∗∗*^*p* < 0.01.

Pancreatic cancer cells (PANC-1 and AsPC-1 cells) were treated with 0, 10, 20, or 40 ng/ml IL-9 for 48 h. Elevated PANC-1 and AsPC-1 cell proliferation was correlated with increased IL-9 concentration (Figure 1(a)); however, the apoptosis rate of both PANC-1 and AsPC-1 cells did not exhibit significant changes (*p* > 0.05). In addition, invasion and migration increased significantly following IL-9 treatment (Figures 1(b) and 1(c)).

### 3.2. miR-200a Inhibits Pancreatic Cancer Cell Proliferation and Metastasis

**Figure 2:**
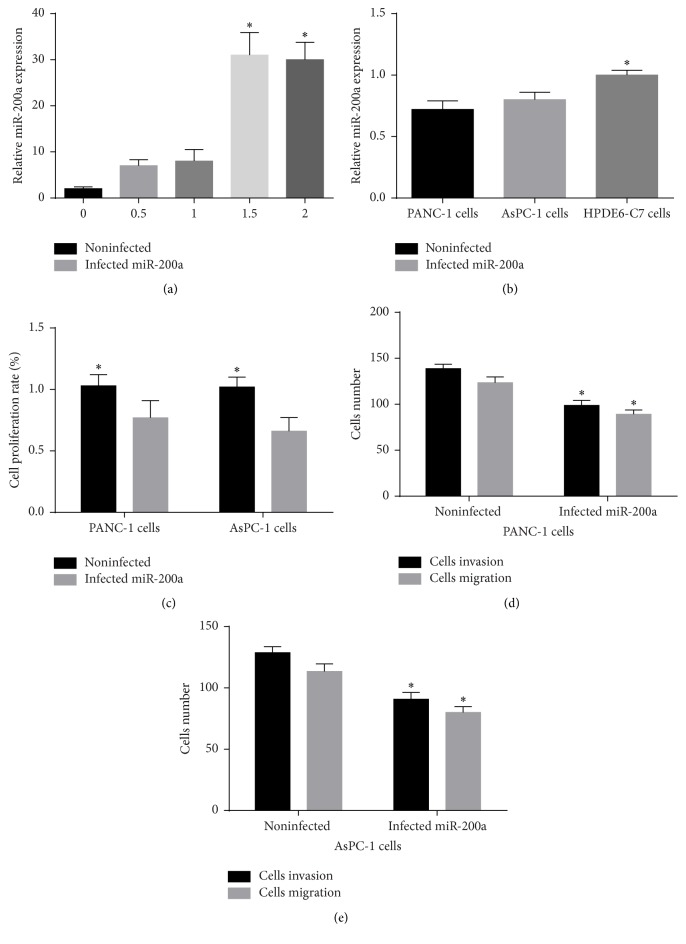
miR-200a inhibited pancreatic cancer cells proliferation and metastasis. (a) The efficiency of lentiviral infection was determined by qRT-PCR at 72 h after infection of miR-200a mimics. (b) Expression of miR-200a in PANC-1, AsPC-1, and HPDE6-C7 cells was tested using qRT-PCR method. (c) Comparison of cells proliferation in between PANC-1 and AsPC-1 cells infected with and without miR-200a mimics using CCK8 method. (d)-(e) Comparison of cells metastasis between PANC-1 and AsPC-1 cells infected with and without miR-200a mimics using Transwell assay. Data was expressed as mean ± SD. ^*∗*^*p* < 0.05.

miR-200a expression was measured in PANC-1 and AsPC-1 cells and pancreatic cells (HPDE6-C7). The expression of miR-200a was increased at least 8 times in a lentivirus-dose-dependent manner (Figure 2(a)). miR-200a expression was lower in PANC-1 and AsPC-1 cells than in HPDE6-C7 cells (Figure 2(b)). Since miR-200a was decreased in pancreatic cancer cells, miR-200a mimics were infected into PANC-1 and AsPC-1 cells. Overexpression of miR-200a in PANC-1 and AsPC-1 cells significantly suppressed cell proliferation, invasion, and migration (Figures 2(c)–2(e)). Collectively, these results indicate that miR-200a expression is downregulated in PANC-1 and AsPC-1 cells and overexpression of miR-200a can inhibit the proliferation and metastasis of PANC-1 and AsPC-1 cells.

### 3.3. *β*-Catenin Is a Direct Target Gene of miR-200a in Pancreatic Cancer Cells

**Figure 3:**
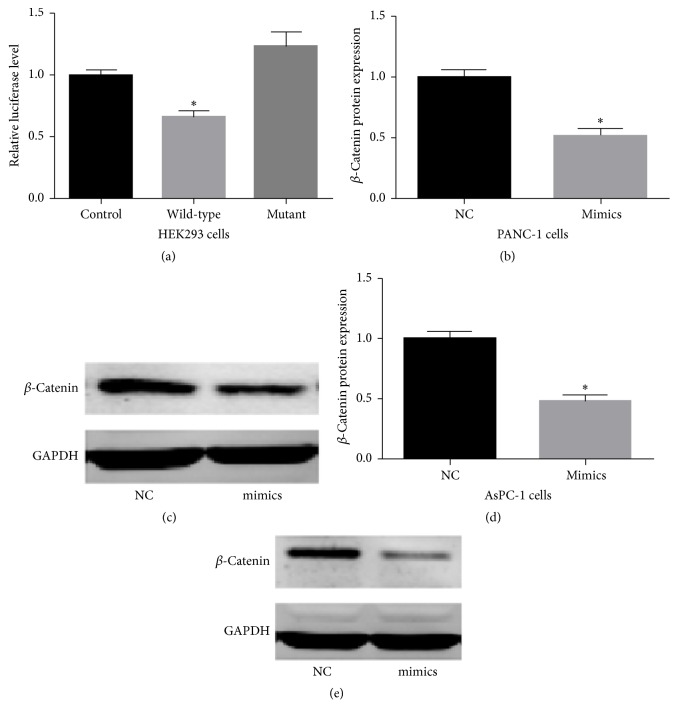
miR-200a target luciferase reporter assay. (a) Reporter construct containing psiCHECK2-WT-*β*-catenin 3′-UTR and psiCHECK2-MUT-*β*-catenin 3′-UTR was cotransfected into HEK293T cells. Relative luciferase activity was normalized to firefly luciferase. Luciferase reporter assay revealed that the relative luciferase level was significantly decreased in cells transfected with miR-200a, suggesting that *β*-catenin was a target of miR-200a. (b) Comparison of *β*-catenin protein levels in miR-200a mimics infection group (miR-200a mimics) and negative control group (NC) in PANC-1 cells; the expression of *β*-catenin protein levels was decreased after infecting miR-200a mimics. (c) Western-blot result of *β*-catenin in PANC-1 cells; GAPDH was used as a control. (d) Comparison of *β*-catenin protein levels in miR-200a mimics infection group (miR-200a mimics) and negative control group (NC) in AsPC-1 cells; the expression of *β*-catenin protein levels was decreased after infecting miR-200a mimics. (e) Western-blot result of *β*-catenin in AsPC-1 cells; GAPDH was used as a control. Data was expressed as mean ± SD. ^*∗*^*p* < 0.05.

Luciferase activity was significantly suppressed in HEK293T cells transfected with wild-type miR-200a compared with cells transfected with the miR-200a mutant (Figure 3(a)). We next infected miR-200a mimics into pancreatic cancer cells and examined the *β*-catenin protein levels by western-blot analysis. *β*-Catenin protein expression was significantly reduced following the infection of miR-200a mimics compared to its expression in noninfected cells (Figures 3(b)–3(e)). These results suggest that *β*-catenin is a direct downstream target gene of miR-200a in PANC-1 and AsPC-1 cells.

### 3.4. IL-9 Promotes Pancreatic Cancer Cells via the miR-200a/*β*-Catenin Axis

**Figure 4:**
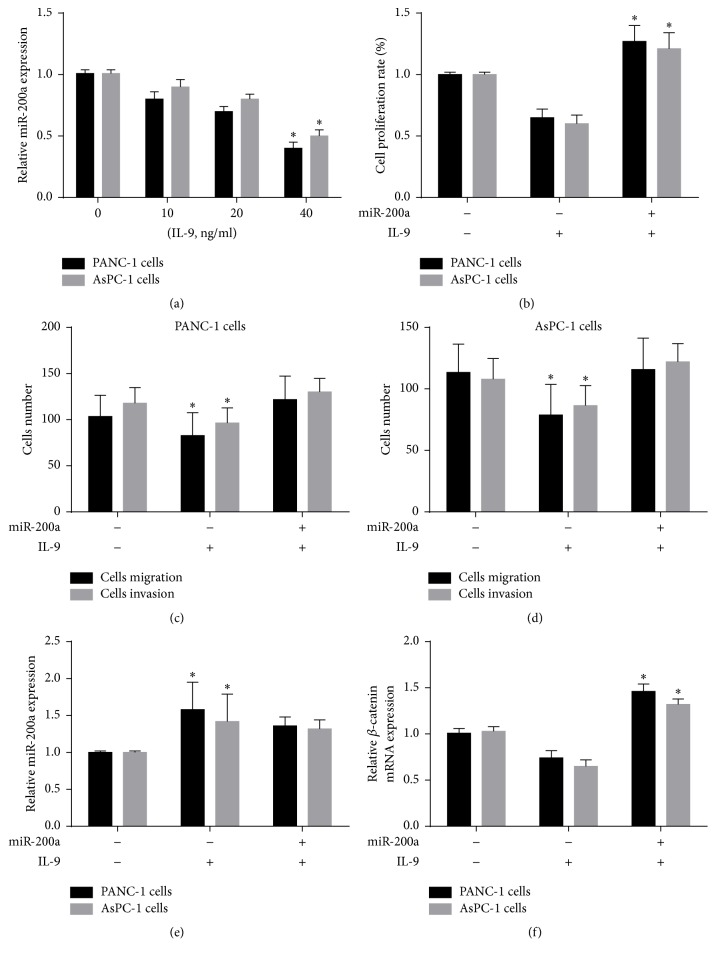
IL-9 promoted pancreatic cancer cells via miR-200a/*β*-catenin axis. (a) PANC-1 and AsPC-1 cells were treated with IL-9 at different concentrations, and the expression of miR-200a was decreased with the elevation of IL-9 concentration. (b) Comparison of PANC-1 and AsPC-1 cells proliferation with the different combination of miR-200a mimics and IL-9. (c)-(d) Comparison of PANC-1 and AsPC-1 cells metastasis with the different combination of miR-200a mimics and IL-9. (e) Comparison of relative miR-200a expression in PANC-1 and AsPC-1 cells with the different combination of miR-200a mimics and IL-9. (f) Comparison of relative *β*-catenin mRNA expression in PANC-1 and AsPC-1 cells with the different combination of miR-200a mimics and IL-9. Data was expressed as mean ± SD. ^*∗*^*p* < 0.05.

In order to examine the interaction between IL-9 and miR-200a in pancreatic cancer cells, PANC-1 and AsPC-1 cells were treated with IL-9 at different concentration, and the expression of miR-200a was decreased with the elevation of IL-9 concentration (Figure 4(a)). Then, the pancreatic cells were infected with miR-200a mimics and then treated by IL-9. PANC-1 and AsPC-1 cell proliferation was enhanced following IL-9 treatment compared to nontreated cells. Similar results were obtained for the invasion and migration of PANC-1 and AsPC-1 cells (Figures 4(b) and 4(c)). In addition, miR-200a expression was decreased and *β*-catenin expression was increased in the miR-200a mimics + IL-9 group compared with the IL-9 treatment group (*p* < 0.05) (Figures 4(d)–4(f)). Taken together, these results indicate that IL-9 might promote PANC-1 and AsPC-1 cell activity via regulation of the miR-200a/*β*-catenin axis.

## 4. Discussion

In the present study, we show that IL-9 promotes pancreatic cancer cell proliferation and metastasis and that this effect might occur via the miR-200a/*β*-catenin axis. In addition, we also observed that IL-9 has a little effect on pancreatic cancer cell apoptosis. In line with a previous study [15], we found that miR-200a expression was downregulated in pancreatic cancer cells compared with normal pancreatic cells and that the overexpression of miR-200a could inhibit proliferation and metastasis in pancreatic cancer cells. Similar to the results obtained in colon cancer cells [16], *β*-catenin was verified as a direct target of miR-200a in pancreatic cancer cells. Treatment of pancreatic cancer cells with IL-9 decreased miR-200a expression and increased *β*-catenin expression; the effect of miR-200a on pancreatic cancer cells was reduced by IL-9 treatment. Our results provide preliminary information regarding the role of the interaction between IL-9 and miR-200a in the regulation of pancreatic cancer cells.

The molecular mechanism underlying pancreatic cancer is complex; several studies have demonstrated that both traditional genes and miRNAs contribute to tumorigenesis in the pancreas. Several miRNAs have been found to be associated with the development and progression of pancreatic cancer. Of these, miR-200a has been shown to be a critical factor able to suppress proliferation and metastasis of some cancers including non-small-cell lung cancer [17], pancreatic cancer [5], and hepatocellular carcinoma [6]. A recent study has identified *β*-catenin as a direct target of miR-200a and that overexpression of M2-type pyruvate kinase negatively regulates *β*-catenin through miR-200a in colon cancer cells [16]. Another study found that downregulation of miR-200a can induce EMT phenotypes and CSC-like signatures by targeting the *β*-catenin pathway in hepatic oval cells [18]. These studies indicate that the miR-200a/*β*-catenin axis may be involved in the proliferation and metastasis of several cell types.

IL-9 has been implicated in a number of immune or inflammatory diseases such as parasitic infection, allergy, and asthma disease [9–11]; however, the function of IL-9 in tumor immunity remains unclear and controversial [19, 20]. Huang et al. [21] reported that IL-9 expression was decreased in the tissue and plasma samples of colon cancer patients and that the decreased expression of IL-9 was correlated with colon cancer progression. However, Hoelzinger et al. [22] showed that eliminating endogenous IL-9 enabled sensitization of host T-cells to tumors, leading to their early rejection without the requirement of vaccines or immunomodulatory therapies. These discrepancies may be partly explained by the timing of IL-9 secretion in a given pathologic circumstance and by the different cell types (such as Th17 and Th9 cells) that express the IL-9 receptor [22]; a number of studies have shown that IL-9 secreted by Th17 or Th9 cells has an opposite effect on experimental autoimmune encephalitis [23, 24].

In this study, we observed that IL-9 was able to promote the proliferation, invasion, and migration of pancreatic cancer cells in a concentration-dependent manner and the miR-200a expression was lower in pancreatic cancer cells than normal pancreatic cells, suggesting that IL-9 has tumor-promoting activities in pancreatic cancer. Thus, anti-IL-9 could be used as a novel potential approach in the treatment of pancreatic cancer metastasis. In addition, we examined the interaction of IL-9 and miR-200a in PANC-1 cells. The results showed that the effect of inhibition of miR-200a on pancreatic cancer cells was promoted following IL-9 treatment. Moreover, IL-9 decreased miR-200a expression and increased *β*-catenin expression even following miR-200a mimics infection, suggesting that the effect of IL-9 on pancreatic cancer cells may occur through the miR-200a/*β*-catenin axis.

A number of study limitations need to be noted. First, only the PANC-1 and AsPC-1 cells lines were used to explore the effect of IL-9 on pancreatic cancer cells and the interaction between IL-9 and miR-200a. These results need to be confirmed using clinical tissues; however, PDAC clinical tissues were unavailable. Second, miR-200b and miR-200c, which belong to the miR-200 family, have been reported to play a key role in the regulation of the EMT process, indicating that miR-200b and miR-200c might play a similar role to miR-200a; however, their role was not examined in this study. Third, although we observed that there was an interaction between IL-9 and miR-200a, the interaction mechanism remains unknown. Fourth, miR-200a was overexpressed in most of our study using miR-200a mimics, but knockdown/knockout miR-200a in pancreatic cells can make the results more reliable. Therefore, future studies should address and resolve these limitations.

In conclusion, our study demonstrates that IL-9 promotes proliferation and metastasis in pancreatic cancer cells and that this effect may occur via regulation of the miR-200a/*β*-catenin axis, suggesting that both IL-9 and miR-200a could be used as potential biomarkers in the treatment of pancreatic cancer.
